# Optimization of a targeted metabolomics kit for dried blood spots analysis and longitudinal comparison with serum

**DOI:** 10.1007/s11306-026-02433-5

**Published:** 2026-04-29

**Authors:** Eleonora Bossi, Cornelia Prehn, Lia Crotti, Gabriella Malfatto, Giuseppe Paglia, Michael Witting

**Affiliations:** 1 Department of Medicine and Surgery, University of Milano-Bicocca, 20900 Monza, Italy; 2 Metabolomics and Proteomics Core, Helmholtz Zentrum München, Ingolstädter Landstraße 1, 85764 Neuherberg, Germany; 3 Department of Cardiology, Cardiac Rehabilitation Unit, IRCCS, Istituto Auxologico Italiano, S. Luca Hospital, Piazzale Brescia 20, 20149 Milan, Italy; 4 TUM School of Life Sciences, Technical University of Munich, Maximus-von-Imhof-Forum 2, 85354 Freising-Weihenstephan, Germany

**Keywords:** Dried blood spots, Targeted metabolomics, Method optimization, LC-MS/MS

## Abstract

Targeted metabolomics kit-based assays are widely used for quantitative metabolic profiling in clinical research. However, they are primarily validated for conventional matrices such as plasma and serum. Their direct application to dried blood spots (DBS) cannot be assumed and requires specific optimization due to intrinsic matrix-specific differences. This study aims to optimize the TMIC MTX MEGA assay for DBS analysis and matrix comparison. Validation was then conducted using paired longitudinal DBS and serum samples from 11 participants in a clinical study undergoing cardiac rehabilitation. A total of 323 compounds were quantitatively measured in DBS and 496 in serum. Principal component analysis revealed matrix-driven separation, highlighting clear distinctions of plasma/serum from DBS and confirming intrinsic matrix differences. Despite global differences, longitudinal trends across metabolite classes and at individual metabolite levels were largely concordant between DBS and serum. Class-specific differences were observed, particularly among lipid species and metabolites influenced by intracellular contributions, consistent with known biological factors. Although absolute concentrations differed for several metabolites, relative temporal changes were preserved. This proof-of-concept study demonstrates the feasibility of applying targeted metabolomics kit-based assays to DBS following workflow optimization. Overall, the optimized DBS workflow shows promise for longitudinal metabolic profiling in clinical contexts. These preliminary findings support further evaluation of DBS as a minimally invasive sampling alternative for targeted metabolomics applications in larger cohorts.

**Graphical abstract:**

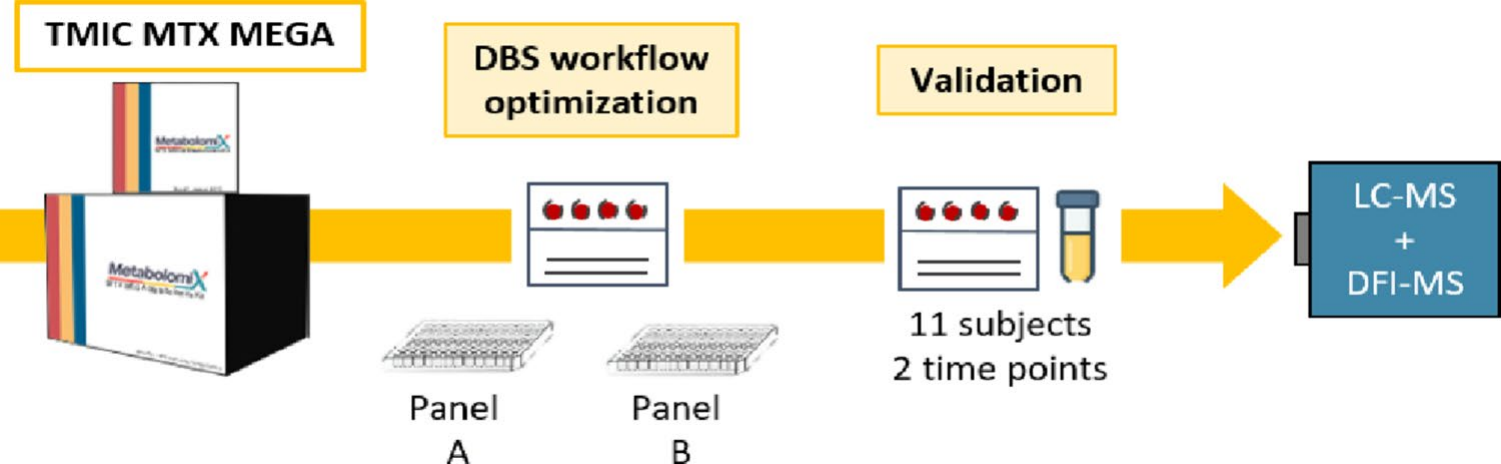

**Supplementary Information:**

The online version contains supplementary material available at 10.1007/s11306-026-02433-5.

## Introduction

Since its initial development in the 1960s for newborn screening, dried blood spots (DBS) and, more generally, blood microsampling have emerged as attractive sampling techniques in research. Compared to traditional venipuncture, the current gold standard, blood microsampling is minimally invasive, requires a lower blood volume, and allows self- and remote sampling without the need for a trained phlebotomist and a strict cold chain for transportation (Couacault et al. 2024). It is considered a viable alternative to traditional sampling, with applications ranging from diagnostics to drug testing, forensic toxicology, and exposure detection (Bossi et al. 2024, Couacault and Witting 2026). In addition, blood microsampling techniques have been successfully applied to animal models to enable minimally invasive serial sampling, reducing animal use (Volani et al. 2018). Considering these characteristics and compatibility with several bioanalytical techniques, such as mass spectrometry (MS)-based platforms, DBS has gained interest and become widespread in clinical and longitudinal studies (Bossi et al. 2025, DeBalsi et al. 2025, Bossi et al. 2026). Blood-based samples are considered the best and most studied biofluids for metabolomics (Kirwan et al. 2018). However, serum and plasma preparations require trained phlebotomists and laboratory personnel, as well as stringent storage conditions, to prevent potential metabolite degradation (Li et al. 2020). As a whole-blood-based matrix, DBS retains the blood cellular fraction, including erythrocytes, white blood cells, and platelets. Previous studies showed that whole blood contains more analytes than serum or plasma, suggesting it better represents a greater portion of the blood metabolome (Stringer et al. 2015, Volani et al. 2023). Moreover, whole blood minimizes the risk of hemolysis and includes intracellular metabolites from blood cells, allowing a more accurate representation of metabolic pathways than serum (Stringer et al. 2015). DBS offers several advantages. Still, it is associated with challenges, including potential hematocrit (HCT) effects when working with non-volumetric devices and preanalytical issues that may complicate comparisons with standard matrices such as serum, plasma, and conventional whole blood. Overall, DBS has been shown to capture broad metabolic profiles, be stable across a range of conditions, and provide representation of metabolome changes comparable to that in plasma or serum, making it suitable for longitudinal metabolic monitoring (DeBalsi et al. 2025). This has enabled metabolomics-based applications across different fields of study, for both untargeted and targeted approaches.

Targeted metabolomics focuses on measuring and quantifying a predefined set of known metabolites using specific analytical or isotope-labeled standards. Although targeted metabolomics is not traditionally used for biomarker discovery, as in untargeted approaches, the ability to quantify known metabolites makes the data useful for comparison with established reference ranges. Therefore, targeted metabolomics data can be used for interpretation in a biomedical context, for clinical research, and for medical diagnosis (Beger et al. 2024, Anh et al. 2024). Research in targeted metabolomics is often performed with commercially available kit-based assays that provide reproducibility through semi-automated, high-throughput, and fully integrated standardized workflows. These assays enable robust, reproducible results and precise metabolite quantification, which are particularly valuable for large cohort studies, inter-laboratory comparisons, and the exchange of data from different studies (Siskos et al. 2017, Paglia et al. 2018).

Additionally, they are usually compatible with a variety of LC-MS/MS platforms (Paglia et al. 2018). Although their use in clinical research is widespread, they are mostly validated in serum, plasma, and other conventional matrices. In addition, to our knowledge, there is very limited DBS-specific literature on the optimization and/or validation of targeted metabolomics assays for DBS (Li et al. 2020). This highlights a methodological gap, as analytical workflows optimized and validated for serum/plasma do not automatically translate to DBS. Therefore, specific optimization strategies are needed when adapting targeted metabolomics assays to DBS.

This study aims to optimize the TMIC MTX MEGA assay specifically for DBS, followed by performance evaluation and comparison with other matrices, including paired serum samples and other reference plasma samples (Zhang et al. 2024). The final analysis focuses on paired DBS and serum samples from subjects participating in a longitudinal clinical study evaluating DBS-derived metabolomic profiles. We detected and quantified 323 metabolites in DBS, 496 in serum, 496 in NIST SRM 1950 plasma, 503 in reference plasma human M18, 453 in reference plasma mouse, and 447 in reference serum human. Our results show that the TMIC MTX MEGA kit can be readily applied to DBS samples.

## Materials and methods

### Chemicals

All extraction solutions and UHPLC solvents were purchased from Merck KGaA (Darmstadt, Germany): water (H_2_O), methanol (MeOH), and acetonitrile (ACN) were LC–MS grade, LiChrosolv^®^, and ethanol (EtOH) was of HPLC-grade. Phenylisothiocyanate (PITC), pyridine, ammonium acetate, formic acid, and phosphate-buffered saline (PBS) were purchased from Sigma-Aldrich/Merck (Darmstadt, Germany).

### Sample collection

Sample collection was performed at Istituto Auxologico Italiano, San Luca Hospital in Milan. For this study, a sub-cohort of 11 male subjects was selected from a larger clinical cohort of 25 consecutive subjects who experienced a first non-complicated myocardial infarction. Inclusion criteria for the original cohort were: male gender, age 25–70 years, and absence of peripheral vascular disease, diabetes mellitus, hemodynamic instability, heart failure (NYHA class II–III), moderate-to-severe chronic obstructive pulmonary disease, or inability to provide informed consent. The cardiac rehabilitation protocol lasted five to seven weeks and consisted of three to five sessions per week (90 min each), for a total of 22 ± 2 sessions per subject. Whole blood samples for DBS, and paired serum samples were collected at two time points: time point A (baseline, before entering the cardiac rehabilitation protocol and time point F (final assessment, at the end of the protocol) (Bossi et al. 2026). This resulted in 22 DBS samples and 22 paired serum samples. Time point A was intended to be collected approximately one week after hospital admission. However, exact timing may have varied across patients due to clinical and logistical factors. Sampling at time point F was performed consistently 15 min before the last training session. DBS were collected on Whatman 903 Protein Saver Cards (Cytiva, Global, Little Chalfont, UK), dried at room temperature (RT) for 2 h, and stored with the corresponding serum samples at -80 °C. HCT was not measured, nor were DBS samples corrected for HCT-related effects. The potential impact is further discussed in the Limitations paragraph. For workflow optimization, blood was collected from the same subject without anticoagulants, and 50 µL were quickly spotted onto Whatman cards to obtain DBS, ensuring consistency and comparability across tested conditions.

### Targeted metabolomics assay

Targeted metabolomics analysis was performed using the TMIC MTX MEGA assay. This targeted MS-based approach enables analyte identification and quantification across major metabolic pathways via a combination of reverse-phase LC-MS/MS and direct injection-MS (DFI-MS) (Zhang et al. 2024). The assay is originally validated for plasma and serum and was adapted and optimized here for DBS analysis.

### Sample extraction

DBS extraction was initially performed according to the manufacturer’s original protocol (Supplementary S1). Following preliminary testing, the protocol was optimized to improve extraction efficiency and analytical performance specifically for DBS, with a focus on increasing metabolite coverage. Finally, it was applied to the analysis of DBS and serum clinical samples, as well as to plasma and serum references for matrix comparison.

DBS were punched (1 × 3 mm) from the Whatman cards and transferred either into the filter plate (Panel A) or into 1.5 mL SafeLock tubes (Panel B). To prevent cross-contamination, the tweezers and the hand puncher were cleaned with ethanol, and three blank punches were performed on clean filter paper between consecutive samples. Calibration and quality control (QC) standards, extraction and derivatization solutions, and DFI buffer were prepared according to the manufacturer’s instructions (See Supplementary S1).

The Panel A workflow (See Supplementary S1) was slightly modified in accordance with our established in-house protocol (Zukunft et al. 2013). A plate-washing step with 100% MeOH was performed before starting the extraction, as standard practice to minimize background signal and interferences. The filter plate was then centrifuged and dried with N_2_ using a TurboVap^®^ 96 Dual plate evaporator (Biotage Sweden AB, Uppsala, Sweden). Internal standards were added to all wells, except the double blanks, as described. First, 10 µL of ‘Panel A - FIS 1’ and 10 µL of ‘Panel A - FIS 2’ solutions were added to each applicable well and dried under N_2_ at RT for 5 min at 5 liters per minute (LPM). Then, 10 µL of ‘Panel A - FS1’ and 10 µL of ‘Panel A - FS2’ solutions were added and dried under identical conditions, followed by 10 µL of ‘Panel A – LIS’. 10 µL each of PBS, calibration standards, QCs, and reference plasma and serum samples were added to the plate according to the plate configuration. The plate was dried under N_2_ at RT for 30 min at 5 LPM. Metabolite extraction was performed by adding 300 µL of extraction solvent to each well, shaking at RT for 30 min at 180 rpm using a MultiShaker (neoLab, Heidelberg, Germany), and centrifuging at RT for 3 min at 500 g. 20 µL aliquots were transferred from each well of the capture plate to the ‘Panel A - LC-MS/MS’ plate and diluted in 180 µL of water, then shaken at RT for 15 min at 300 rpm. The DFI-MS/MS plate was prepared as follows. 10 µL were transferred from the Panel A capture plate to the ‘Panel A – DFI-MS/MS’ plate, diluted with 490 µL of DFI buffer, and shaken at RT for 15 min at 300 rpm.

The Panel B extraction workflow (See Supplementary S1) was specifically optimized for DBS by testing 12 different combinations of extraction solutions and conditions in triplicate (Table 1). The optimal protocol was selected based on metabolite coverage. A detailed step-by-step comparison between the original and optimized protocols is provided in Supplementary Table S1b.

**Table 1:** Panel B extraction DBS optimization – The present table describes the condition names (condition ID), the DBS punch size in mm, and quantity of punches, the extraction solutions (See Step e Supplementary S1), and the extraction conditions (See Step f Supplementary S1) that were tested for the optimization of Panel B

Condition ID	DBS punch size (mm)	Extraction solution	Extraction condition
C1 (original protocol)	1 × 3	90 µL 100%MeOH	-20 °C overnight incubation
C2	1 × 3	90 µL 100%MeOH	20 min shaking at 4 °C
C3	1 × 3	90 µL 75%MeOH	20 min shaking at 4 °C
C4	1 × 3	90 µL 75%MeOH	20 min shaking at 4 °C, ultrasonic bath
C5	1 × 3	70 µL 75%MeOH	20 min shaking 4 °C
C6	2 × 3	90 µL 75%MeOH	20 min shaking 4 °C
C7	1 × 3	90 µL 75%MeOH	20 min shaking 4 °C, 5 µL IS
C8	1 × 3	22.5 µL 50%MeOH + 67.5µL 100%MeOH	-20 °C overnight incubation
C9	1 × 3	22.5 µL H2O + 67.5µL 100%MeOH	-20 °C overnight incubation
C10	1 × 3	22.5 µL H2O + 67.5µL 100%MeOH	20 min shaking 4 °C
C11	1 × 3	17.5 µL H2O + 52.5µL 100%MeOH	20 min shaking 4 °C
C12	1 × 3	22.5 µL PBS + 67.5µL 100%MeOH	20 min shaking 4 °C

For the final analysis comparing DBS and serum samples, the sample preparation was performed as follows. In Panel B, pooled samples were first generated by mixing 10 µL of each sample: a serum pool from all serum samples and a DBS pool from all DBS supernatants after centrifugation. Both pools were subsequently processed in Panel A by loading them onto the filter plate. Finally, the DFI plate was prepared from Panel A. DBS and serum samples were analyzed in single due to limited DBS availability (only 1 × 3 mm punch per time point in different subjects). DBS and serum pool samples were added in triplicate to both panels to assess analytical consistency. To enable matrix comparison, different reference materials were included in both panels and were also used in the optimization phase: NIST SRM 1950 plasma (triplicate), reference plasma human M18 (10 times), reference plasma mouse (triplicate), and reference serum human (5 times). Reference plasma human M18 was purchased from SeraLab (BioIVT, West Sussex, UK, Prod. Number: HUMANPLK3PNN). QC2 was added four additional times across the plate to monitor performance throughout the analysis.

### LC-MS methods

Samples were analyzed using an LC-MS platform comprising a Shimadzu LC-30AD (Shimadzu Corporation, Kyoto, Japan) liquid chromatography system coupled to a QTRAP 5500 mass spectrometer (SCIEX, Marlborough, MA, USA) operating in multiple reaction monitoring (MRM) mode. Chromatographic separation was achieved using a Zorbax Eclipse XDB C18 (3.0 × 100 mm, 3.5 μm) column (Agilent Technologies, Palo Alto, CA, USA) with a Phenomenex C18 (4 × 3 mm) guard column (Phenomenex, Torrance, CA, USA), following the TMIC MTX MEGA instructions, LC-MS and DFI-MS methods (Zhang et al. 2024). Only the LC-MS injection volumes were modified during method optimization for DBS: 15 µL for Panel A and 20 µL for Panel B.

### Data analysis

Analyst^®^ software (SCIEX, Marlborough, MA, USA) was used for data acquisition. Data processing was performed using the integrated LC-AutoFit web application and MultiQuant™ (SCIEX, Marlborough, MA, USA) for calibration, peak integration, and quantification. Statistical analyses were performed using R 4.5.1 (R Foundation for Statistical Computing, Vienna, Austria) and GraphPad Prism 9.5 (GraphPad Software, Boston, MA, USA, www.graphpad.com). Concentrations below the limit of detection (LOD) were treated as missing values. Metabolites with more than 50% missing values across the dataset were excluded from further analysis. The remaining missing values were imputed using the half-minimum method within each subject-time point group. Data were then normalized using sum normalization and a log_10_ transformation before statistical analysis.

## Results and discussion

### Workflow optimization

The results of this work provide an optimized extraction workflow for the TMIC MTX MEGA assay specifically for DBS. The optimization process started from the manufacturer’s protocol and involved independent optimization of Panel A and Panel B (See Supplementary S2) (Zhang et al. 2024). Panel A was optimized according to an established in-house workflow for targeted metabolomics (Zukunft et al. 2013). In contrast, Panel B was optimized *de novo*: twelve different combinations of extraction solutions and conditions (Table 1) were tested and compared to the original protocol.

**Fig. 1:**
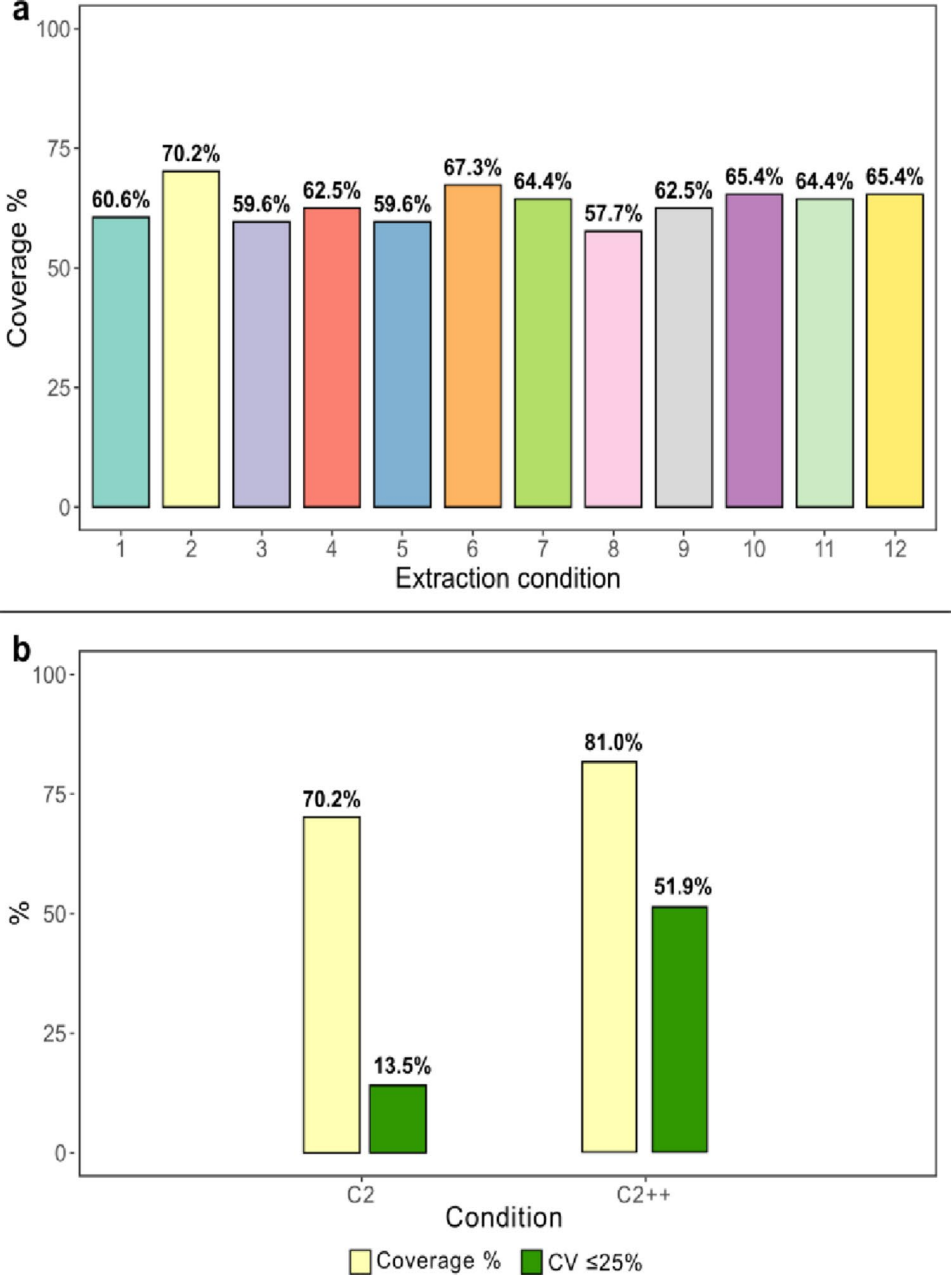
**a** Panel B workflow optimization - Metabolite coverage. The barplot shows metabolite coverage (%) on the Y-axis, measured as the number of metabolites above LOD relative to the total number of metabolites detected. It is expressed as a percentage per condition (X-axis). C1 is the original protocol. The other conditions are summarized in Table 1. **b** Results of the optimization of condition C2 expressed in terms of coverage % (light yellow) and % of metabolites with CV ≤ 25% (green). Bars on the left represent C2 before optimization. Bars on the right (C2++) represent C2 after optimization

For each metabolite above the LOD, the mean value for each condition was divided by the mean value of the same metabolite from the original protocol (C1). Ratios greater than 1 were assigned a value of 1, whereas ratios ≤ 1 were assigned a value of 0. Considering the sum of these binary values per condition, C2 (90 µL 100% MeOH and 20 min shaking at 4 °C) and C6 (2 × 3 mm punches, 90 µL 75% MeOH and 20 min shaking at 4 °C) resulted in the highest total scores. Consistent results were obtained when considering metabolite coverage per condition, defined as the number of metabolites detected above the LOD out of the total metabolites detected in Panel B, expressed as a percentage (Fig. 1a).

C6 was not further considered because it included 2 × 3 mm DBS punches, and for the validation analysis with clinical samples, only a 1 × 3 mm punch was available for some patients. Therefore, C2 was selected as the new condition for further optimization. To further increase metabolite coverage, the final sample dilution was changed from the original 1:10 to 1:5, as DBS samples were likely too diluted due to the lower blood volume. The LC-MS injection volume was also increased to 20 µL for the same reason: to improve detection of metabolites slightly below the LOD. This second optimization step, performed directly on C2, not only improved metabolome coverage from 70.2% to 81.0%, but also increased the number of metabolites with a CV below 25% from 13.5% to 51.9% (Fig. 1b).

Based on these results, the extraction protocol was optimized for DBS by replacing the − 20 °C overnight incubation with shaking at 4 °C for 20 min, while keeping 100% MeOH as the extraction solvent, as in the original protocol. Additionally, the dilution factor was modified from 1:10 to 1:5, and the LC-MS injection volume was increased from 10 µL to 20 µL.

### Matrix comparison

The optimized workflow was then applied to matrix comparison analysis to evaluate DBS assay performance relative to the paired serum pool and other reference matrices.

**Fig. 2:**
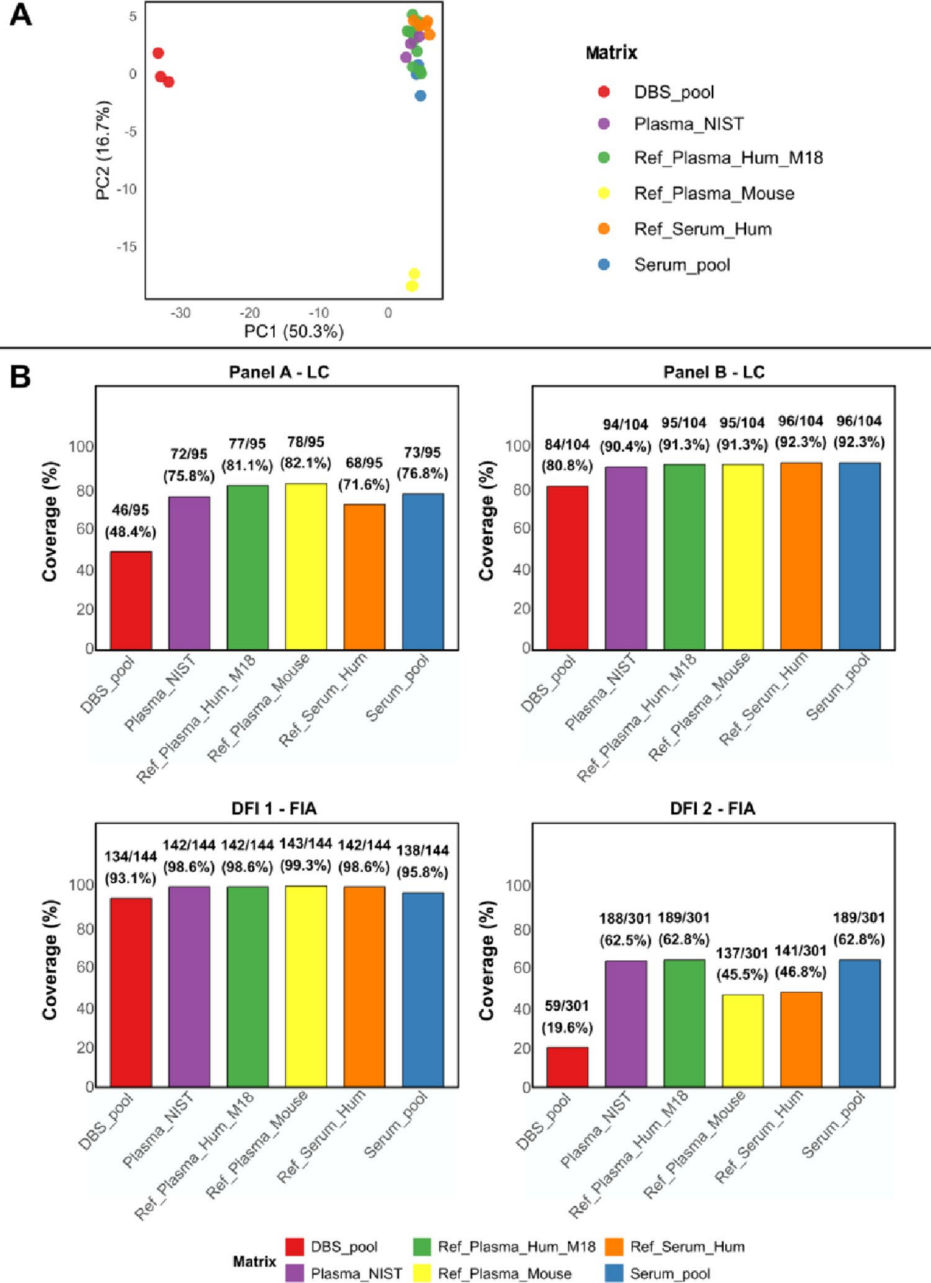
**a** Principal component analysis on auto-scaled data, scores plot of the different matrices included in the analysis. Scores plot of PC 1 and PC 2: points are colored by matrix type, and percentages indicate variance explained by each component. PC1 and PC2 captured the major sources of variance related to the sample matrix. **b** Metabolite coverage per panel and injection mode. Metabolite coverage is expressed both as a percentage and as the number of metabolites detected above the LOD relative to the total number of metabolites detected in each panel or injection mode and in each matrix

Principal component analysis (PCA), performed on auto-scaled data showed that the first component (PC1) clearly grouped DBS pool separately from the serum pool, reference human plasma, reference serum, and NIST SRM 1950 plasma (Fig. 2a). Reference mouse plasma was instead clustered separately due to the variance of the second component (PC2) from both groups. This highlights the differences between the matrices: DBS differs from plasma and serum samples, and plasma samples from mice involve a different species. Therefore, DBS is metabolically distinct from serum/plasma, as it is whole blood. In particular, DBS samples contain cellular components, including red blood cells (RBC), which contribute intracellular metabolites that are absent in serum. In addition, lipid profiles may be influenced by the presence of cellular membranes, leading to systematic differences between matrices. These aspects are further discussed in the context of longitudinal paired samples.

Considering the metabolite coverage per panel (A vs. B) and injection mode (LC vs. DFI) (Fig. 2b), overall, as expected, DBS metabolite coverage is lower compared to plasma and serum, given the lower total blood volume, potential HCT-effect of filter paper, and intrinsic differences of whole blood compared to plasma and serum. Results for Panel A show a difference in metabolite coverage of around 30% compared to the serum pool and the other matrices. For Panel B, the coverage performance was significantly better for DBS, with a metabolite coverage of 80.8% compared to an average of 91.3% for the serum pool and reference matrices. Regarding FIA, DFI 1 showed great performance for all matrices (around 98.6%), 95.8% for the serum pool, and a close 93.1% for the DBS pool. In contrast, results for DFI 2 were lower across all matrices. The highest coverage was obtained for plasma NIST, reference human plasma, and serum pool (62.8%). Lower coverage was observed in reference mouse plasma and reference human serum (46%), whereas in the DBS pool, it was the lowest (< 20%).

### Paired DBS-serum comparison

**Fig. 3:**
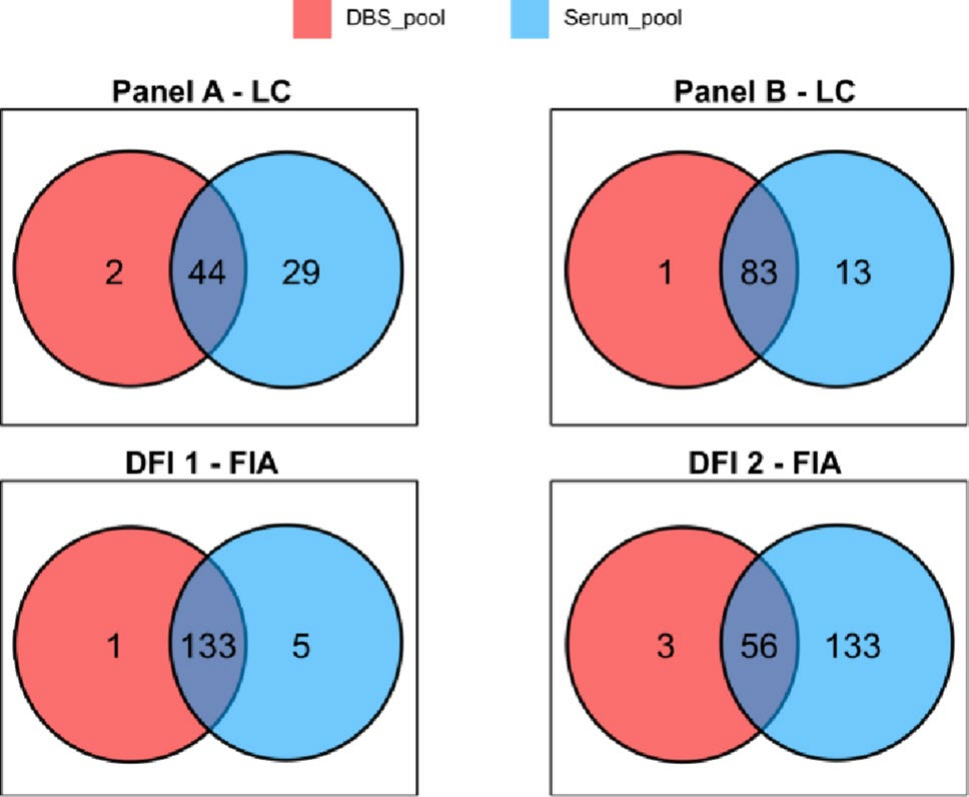
Venn diagrams: metabolite overlap DBS pool (red) vs. Serum pool (blue) per panel and injection mode. It describes the number of detected metabolites exclusive to DBS or serum, and those detected in both matrices

The optimized workflow was applied to the analysis of paired DBS and serum samples from 11 subjects enrolled in a cardiac rehabilitation study. In total, 323 compounds were quantitatively measured in DBS and 496 in serum, all above the LOD. To characterize matrix-dependent metabolite detectability, Venn diagrams were generated for DBS pool and serum pool across Panel A, Panel B, DFI 1, and DFI 2 (Fig. 3). A substantial number of metabolites were detected in both DBS and serum across all analytical panels, indicating broad analytical overlap between matrices. However, matrix-specific compounds were also observed, with a predominance of serum-exclusives in DFI 2, where 133 metabolites were detected. DFI 2 covers lipid classes such as ceramides, cholesterol esters, hexosylceramides, dihexosylceramides, trihexosylceramides, diacylglycerols, and triacylglycerols. The greater number of serum-exclusive detected metabolites within DFI 2 likely reflects matrix-dependent differences in lipid distribution and analytical performance, potentially contributing to differences in metabolite detection and quantification between matrices.

**Fig. 4:**
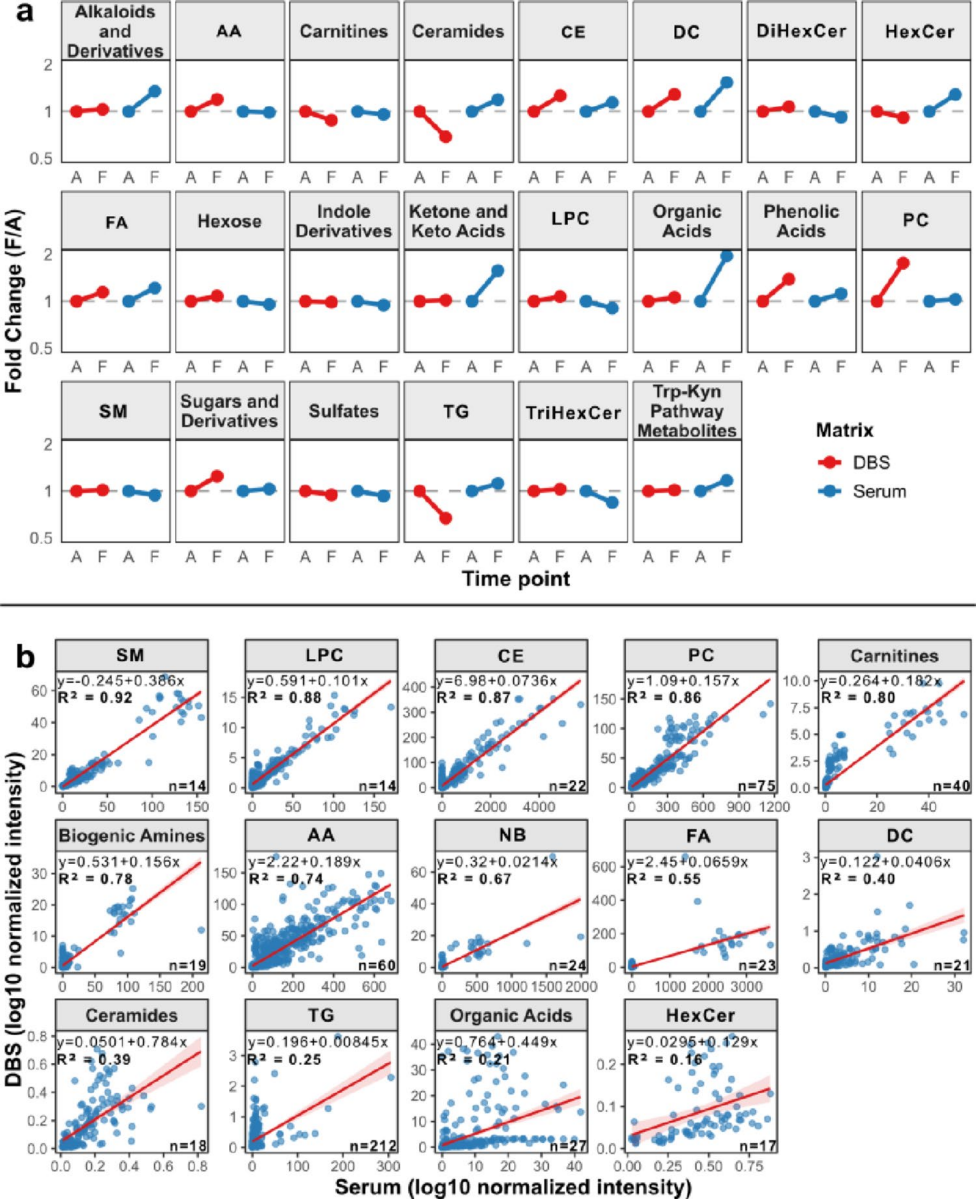
Metabolite class level DBS-serum concordance. **a** FC (F/A) trends by metabolite classes, highlighting similarities or differences in trends between DBS (red) and serum (blue) samples in the comparison between timepoint A and F. Punctual classes were excluded. **b** Pearson correlation between log_10_-transformed concentrations in DBS and paired serum for each metabolite class. Only classes with ≥ 10 paired observations (DBS and serum from the same patient and timepoint) were included. Furthermore, a threshold of at least 10 detected metabolites per class was chosen to ensure robust, representative correlations. Linear regression lines (red) were fitted using ordinary least squares, and the coefficient R^2^ was calculated to quantify the proportion of variance explained. Points represent individual patient-timepoint pairs. The number of metabolites detected in that class is specified on the bottom right of each class plot. AA: Amino acids and derivatives; CE: Cholesterol esters; DC: Diacylglycerols; DiHexCer: Dihexosylceramides; HexCer: Hexosylceramides; FA: Fatty acids and derivatives; LPC: Lysophosphatidylcholines; PC: Phosphatidylcholines; SM: Sphingomyelins; TG: Triacylglycerols; TriHexCer: Trihexosylceramides; NB: Nucleobases and nucleosides

After establishing panel-specific differences in metabolite coverage, we next focused on the metabolite-class level. By considering median fold change (FC) trends by class, we observed similar trends across several classes in both matrices, as well as some opposite trends (Fig. 4a). The largest differences were observed in ceramides and triacylglycerols, with decreasing trends in DBS and increasing trends in serum. Notably, these classes are covered by DFI 2, which showed lower metabolite coverage in DBS (Fig. 2b) and a greater number of serum-exclusive detected metabolites (Fig. 3). Overall, several metabolite classes showed concordant FC trends between DBS and serum. Pearson correlation analysis was then performed to further evaluate the agreement between DBS and paired serum concentrations for each metabolite class (Fig. 4a). The analysis was conducted at the subject-time point level, with correlation coefficients calculated for each pair of DBS and serum samples from the same patient at the same time point. Only classes with at least 10 paired observations were considered for the analysis. A correlation coefficient R² ≥ 0.70 was considered indicative of acceptable agreement between matrices, consistent with previous studies comparing DBS and plasma/serum in targeted metabolomics (Shi et al. 2024) (See Supplementary S6 for correlation coefficients). The correlation plot shows that several classes of metabolites, such as sphingomyelins, lysophosphatidylcholines, cholesterol esters, phosphatidylcholines, carnitines, biogenic amines, and amino acids and derivatives, correlate well, with R^2^ ≥ 0.70. Classes such as nucleobases and nucleosides, fatty acids and derivatives, and diacylglycerols showed moderate correlations (0.40 ≤ R^2^ < 0.70). In contrast, weaker correlations (R^2^ < 0.40) were observed in classes likely to have higher biological variability or lower abundance (e.g., ceramides, triacylglycerols, organic acids, and hexosylceramides). Regarding triacylglycerols, DBS contains more phospholipids and fewer triacylglycerols compared to serum (Gao et al. 2017). For ceramides and hexosylceramides, previous studies have shown higher concentrations in whole blood than in serum (Wang et al. 2021, Meikopoulos et al. 2023). The results showed some differences between DBS and serum, as expected, and likely reflecting the intrinsic differences in their compositions. DBS is a whole-blood-based matrix that includes red blood cell (RBC) components, which are removed during blood processing to obtain serum and plasma samples (Zubkowski et al. 2025, Chiu et al. 2024). As a result, metabolites that are abundant within cells may exhibit different relative changes in DBS compared to serum. Therefore, RBC-abundant classes of metabolites are expected to be higher in whole blood than in serum. In addition to biological factors, matrix-specific analytical effects may also contribute to the observed differences. These include drying, paper retention, potential HCT-related effects, and extraction efficiency. Previous studies have shown that only a subset of metabolites exhibit strong correlations between DBS and plasma/serum, and that lipid class distributions can vary across matrices, likely reflecting matrix-intrinsic and preanalytical differences (Tobin et al. 2021). Despite these differences, the overall high correlation observed in this study suggests a robust relationship between DBS and serum (Shi et al. 2024).

**Fig. 5:**
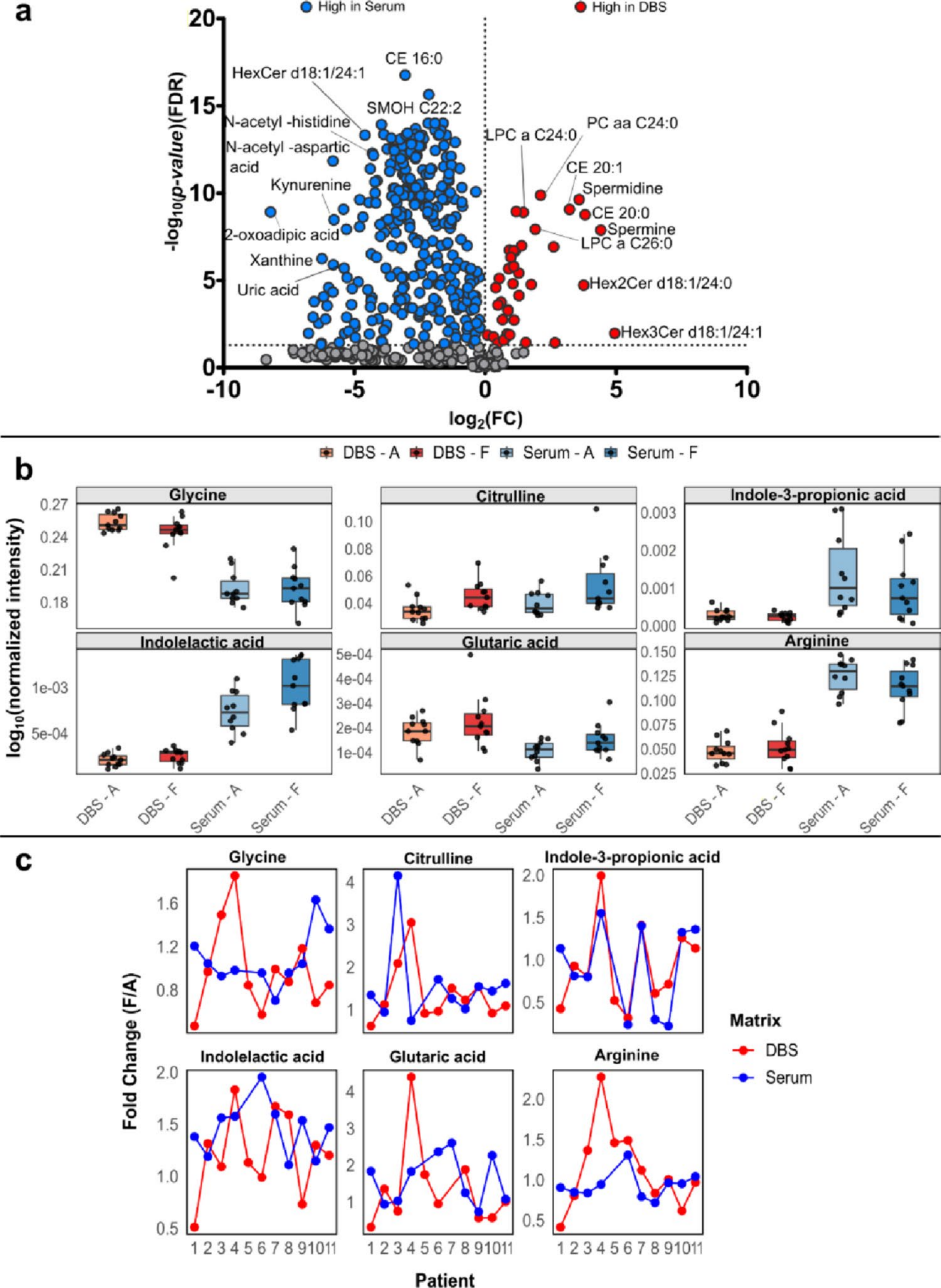
Metabolite-class level analysis. **a** Volcano plot showing paired t-test analysis with FDR correction, 0.05 threshold. Blue dots represent metabolites with higher concentration in serum. Red dots indicate metabolites with higher concentration in DBS. **b** Boxplot of selected significant metabolites in paired t-test analysis DBS and serum timepoint A vs. F. log_10_ (normalized concentration) is on the y-axis. **c** FC (F/A) trend plots for the same selected significant metabolites (as panel B), across all subjects in both DBS (red) and serum (blue). Dots represent individual patients. Dots for patient 5 are missing, as across these selected metabolites, concentrations at timepoint A < LOD; therefore, it was not possible to calculate the FC for this subject

Following the class-level comparison, metabolite-level differences between the two matrices were further explored. A paired comparison between DBS and serum samples was performed using a paired *t*-test with false discovery rate (FDR) correction. Results were visualized as a volcano plot (Fig. 5a). In total, 298 metabolites were found to be significantly different (FDR < 0.05) between the two matrices. Effect sizes and 95% confidence intervals are reported in Supplementary S7. Given the paired study design, the *t*-test was selected for its greater statistical power compared to non-parametric alternatives when the assumption of normality of paired differences was reasonably satisfied, in agreement with previous studies (Meek et al. 2007). The highest number of high-concentration metabolites was observed in serum compared to DBS. This likely reflects a combination of intrinsic matrix-specific differences, potential analytical issues, and the lower volume involved in DBS. Consistent with previous literature, metabolites such as creatine, spermine, and spermidine were significantly increased in DBS, while kynurenine, tryptophan, and free carnitine (C0) were significantly decreased in DBS compared to serum. The significantly lower concentrations of kynurenine in DBS align with previously reported matrix-dependent effects on kynurenine pathway metabolites. Comparative analyses of matched whole blood, serum, and plasma have shown that kynurenine and other tryptophan metabolites tend to be present at higher levels in serum and plasma than in whole blood, potentially reflecting both differential partitioning and differences in matrix composition.

Furthermore, preanalytical factors, including cellular metabolism and matrix stability, may affect the measured abundance of kynurenine pathway intermediates in whole-blood matrices (Heng et al. 2023). These patterns are once again consistent with the different matrix nature, composition, and RBC presence in DBS. RBCs are responsible for minimal tryptophan storage and, as a result, have lower tryptophan concentrations in DBS than in serum (Zubkowski et al. 2025). Overall, these metabolites are more abundant or more stable in the extracellular compartment; therefore, they are relatively reduced in DBS compared to serum. Conversely, creatine and biogenic amines, such as spermine and spermidine, are present at higher concentrations within cells and may therefore appear elevated in DBS than in serum.

To further assess longitudinal changes at the individual metabolite level, an exploratory analysis of single metabolite trends across the 11 patients was performed. Six metabolites showing nominal significance in paired time point A-F comparisons in DBS and serum were selected for detailed evaluation. Boxplots revealed consistent directional changes between A and F in both matrices, despite differences in absolute concentrations and matrix-intrinsic characteristics (Fig. 5b). Fold-change (FC) trend evaluation across subjects showed that, although inter-individual variability and matrix-intrinsic effects were evident, longitudinal trends were somewhat preserved between DBS and serum. For example, FC trends were particularly similar for citrulline and indole-3-propionic acid, with increases or decreases observed in serum mirrored in DBS (Fig. 5c). This suggests that both matrices can consistently capture relative intra-individual changes. Overall, these findings support the potential of this optimized DBS workflow for longitudinal targeted metabolomics analyses, as temporal changes are consistently captured even when absolute concentrations differ from serum. This consistency was observed with the TMIC MTX MEGA assay after DBS optimization in this setting, highlighting the importance of matrix-specific workflow adaptations. A further aspect to consider is the comparability of absolute metabolite concentrations between DBS and serum. In this study, no normalization strategies, matrix-specific calibration curves, or conversion factors were applied to harmonize measurements across matrices. This choice reflects the proof-of-concept nature of this work, primarily aimed at evaluating the feasibility and applicability of a targeted metabolomics kit for DBS by assessing the preservation of longitudinal trends rather than achieving absolute equivalence between matrices.

Overall, following DBS-specific optimization, the TMIC MTX MEGA assay captures longitudinal metabolic trends in DBS that are largely consistent with serum, with class-specific deviations likely associated with intrinsic matrix differences. Therefore, in this study, DBS shows good longitudinal agreement at the relative level when analyzed with targeted metabolomics assays. However, given the limited sample size and the cohort’s homogeneous nature, further validation in larger, more diverse populations is necessary before broader application. Future studies incorporating matrix-specific normalization strategies and evaluating specific conversion factors may further improve the interpretability of DBS data relative to serum measurements, thereby establishing robust frameworks for cross-matrix comparability.

### Limitations

This study presents several limitations that should be considered when interpreting the results. The sample size was small (*n* = 11) and limited to a highly selected sub-cohort of subjects who experienced a first non-complicated myocardial infarction. This small, homogeneous population limits the generalizability of the findings to broader or more diverse groups. A notable limitation of the present work is that the HCT was not measured, and no correction was applied to account for HCT-related variations in DBS. HCT is a well-recognized challenge in DBS analysis, as it can influence spot size, sample homogeneity, and blood viscosity, thereby affecting absolute concentrations, detectability, and recovery of specific metabolites. Although previous studies have reported acceptable analytical performance of DBS even without HCT correction (Li et al. 2020, Torres et al. 2024, Ackermans et al. 2021), the lack of measurements or correction remains a limitation of the present validation work and limits broader generalization. In addition, the time interval between the two sampling time points was not strictly standardized, as it depended on individual rehabilitation protocol and hospital scheduling. Time point A was intended to be collected one week after hospital admission, but the exact timing could not be verified for all patients. It may have varied due to clinical and logistical factors.

In contrast, sample collection at time point F was performed consistently 15 min before the final training session. This variability in sampling time may have introduced additional biological variability. Another limitation is that the analysis of clinical paired samples was performed in a single run due to limited DBS sample availability. Therefore, technical variability and analytical precision could not be directly assessed at the individual sample level. Despite these limitations, the paired design and the optimized workflow enabled the detection of consistent longitudinal trends across matrices in this setting. Future studies, including larger, more diverse cohorts, standardized sampling time, and HCT correction, are warranted to validate the clinical applicability of DBS for targeted metabolomics.

## Conclusion

In the present study, we addressed a methodological gap in targeted metabolomics by optimizing a commercially available kit-based assay specifically for DBS. While the use of targeted metabolomics kits is routine for conventional matrices such as serum and plasma, their direct translation to DBS protocols cannot be assumed due to intrinsic matrix-specific differences. In this work, we tested, adapted, and optimized the TMIC MTX MEGA assay workflow for DBS.

As part of the optimization process, the overall metabolite coverage for Panel B in DBS increased compared to the initial tests, and reproducibility was further improved. We also provided results from matrix comparisons, showing assay performance in terms of metabolite coverage across six matrices and highlighting matrix-specific similarities and differences.

Comparative analyses demonstrated that DBS and serum exhibit distinct global metabolic profiles, as expected. While longitudinal trends at both the metabolite class and individual metabolite levels were largely preserved across matrices, class-specific differences were observed, particularly among triacylglycerols and RBC-related metabolites. In this proof-of-concept cohort, DBS consistently captured relative metabolic changes over time. These findings highlight that, despite differences in absolute concentrations, DBS can provide useful longitudinal metabolic information in this setting when proper workflow optimization is implemented.

Overall, this work supports the feasibility of DBS as a practical and informative matrix for targeted metabolomics in selected longitudinal and clinical settings. Importantly, it underscores the necessity of matrix-specific optimization when applying kit-based assays beyond the originally validated matrices. The optimized DBS workflow presented here expands the applicability of targeted metabolomics, specifically the TMIC MTX MEGA assay, to minimally invasive sampling strategies, facilitating future clinical longitudinal studies in similar settings after proper optimization. As this targeted assay was primarily developed and validated for plasma, its metabolite panel does not comprehensively cover the intracellular metabolome present in DBS. Therefore, while the targeted workflow provides robust quantification of selected metabolites, it does not fully capture intracellular components that may be detectable through untargeted approaches. This represents a methodological limitation when comparing targeted and untargeted DBS approaches. Future development of specific targeted panels that better incorporate intracellular metabolites may further enhance the analytical complementarity of targeted and untargeted workflows.

## Supplementary Information

Below is the link to the electronic supplementary material.

Supplementary Material 1

Supplementary Material 2

Supplementary Material 3

## Data Availability

Raw data and tables reported in this paper are available via [https://zenodo.org/records/18740861](https:/zenodo.org/records/18740861).

## Declarations

The study was conducted in accordance with the principles of the Declaration of Helsinki (2008) and the International Conference on Harmonization Good Clinical Practice guidelines. Written informed consent was obtained from all participants in the present study.
