# Supplementary material for: Optimization of a targeted metabolomics kit for dried blood spots analysis and longitudinal comparison with serum
Source: Metabolomics. 2026 Apr 29;22(3):59. doi: 10.1007/s11306-026-02433-5 (PMC13124823; doi:10.1007/s11306-026-02433-5)
Supplement: Supplementary file 3 — Supplementary Material 3 [file 11306_2026_2433_MOESM3_ESM.docx]

**Supplementary information**

**S1 -** Original TMIC MTX MEGA kit protocol. The following protocol corresponds to the original procedure provided by the manufacturer.

- **Panel A + DFI**
  1. Thaw plasma/serum samples on ice and vortex before use.
  2. Remove the rack from the ‘Panel A – SamplePrep’ filter plate. If any filter does not sit at the bottom of the plate, centrifuge the plate for 2 min at 50 g.
  3. Prepare the calibration and QC standards (See Table S1).
  4. Add 10 μL of both ‘Panel A – FIS1’ and ‘Panel A – FIS2’ solutions to all wells of the filter plate, except for the double blank well position.
  5. Add 10 μL of both ‘Panel A – FS1’ and ‘Panel A – FS2’ solutions to all calibration standards and QC wells.
  6. Dry the plate under N2 at RT for 10 min at 5 LPM.
  7. Add 10 μL of ‘Panel A – LIS’ to all wells except for the double blank well.
  8. Add 10 μL of PBS, calibration standards, QCs and plasma/serum samples according to the plate configuration.
  9. Dry the plate under N2 at RT for 30 min at 5 LPM.
  10. Prepare fresh pre-mix derivatization solution: 1900 μL each of ethanol, pyridine and water. Vortex for 60 seconds.
  11. Prepare 5% PITC solution: add 300 μL of fresh PITC to the pre-mix solution prepared in step j.
  12. Add 50 μL of 5% PITC solution to each well.
  13. Cover the plate with a lid and incubate for 20 min at RT.
  14. Dry the plate under N2 for 90 min at 5 LPM.
  15. Prepare the extraction solvent: dissolve 76 mg of ammonium acetate in 200 mL of MeOH.
  16. Add 300 μL of extraction solvent to each well and shake at 300 rpm at RT for 30 min.
  17. Centrifuge the plate at 50 g for 3 min.
  18. Separate the lower capture plate from the upper filter plate.
  19. Transfer 50 μL from each well of the capture plate to the corresponding wells of the ‘Panel A – LC-MS/MS’ plate. Add 450 μL of water to each well, cover the plate with a silicone mat and shake at 500 rpm for 15 min. Transfer the plate to the LC for analysis.
  20. Prepare DFI buffer: mix 9 mL of ‘DFI Buffer’ with 260 mL of MeOH.
  21. Transfer 10 μL from each well of the capture plate to the corresponding wells of the ‘Panel A – DFI-MS/MS’ plate.
  22. Add 490 μL of DFI buffer to each well of the ‘Panel A – DFI-MS/MS’ plate, cover it with a silicone mat and shake at 500 rpm for 15 min. Transfer the plate to the LC for analysis.
- **Panel B**

1. Thaw plasma/serum samples on ice and vortex before use.
2. Label 1.5 mL Eppendorf tubes for double blank samples, 3 blanks, 7 calibration standards, 3 QCs and plasma/serum samples.
3. Prepare the calibration and QC standards (See Table S1).
4. Add 30 μL of PBS, calibration standards, QCs and plasma/serum samples to the corresponding labeled tubes.
5. Add 90 μL of ice-cold MeOH to each tube for protein precipitation.
6. Vortex each tube for 30 seconds and transfer to a -20°C freezer overnight.
7. Centrifuge all the plasma/serum samples at 18,000 g for 15 min at 4°C.
8. Transfer 50 μL of the double blank, blanks, calibration standards, QCS and plasma/serum sample supernatants to the corresponding wells of the ‘Panel B – SamplePrep’.
9. Prepare Reagent A, Reagent B, Reagent C and Reagent L-RA:
   - 1. Reagent A: 3 mL of MeOH 50% in water to the Reagent A glass vial and vortex until the solid in the vial is fully dissolved.
     2. Reagent B: 3 mL of MeOH to the Reagent B glass vial and vortex until the solid in the vial is fully dissolved.
     3. Reagent C: mix 215 μL of pyridine and 2635 μL of MeOH 75% in water in the Reagent C glass vial and vortex for 30 seconds.
     4. Reagent L-RA: add 25 μL of MeOH 50% in water to the ‘Panel B – L-RA’ tube and vortex for 60 seconds.
10. Transfer 25 μL of Reagent B and Reagent C to the ‘Panel B – L-RA’ tube.
11. Add 50 μL of ‘Panel B – LIS’ solution to the ‘Panel B – L-RA’ tube and vortex.
12. Transfer 2.8 mL each of Reagent A and Reagent B to the Reagent C vial and vortex for 30 seconds.
13. Add 75 μL of the solution prepared in Step l to each well of the ‘Panel B – SamplePrep’ plate.
14. Shake the ‘Panel B – SamplePrep’ plate and the ‘Panel B – L-RA’ tube at 500 rpm for 2 hours at RT.
15. Add 350 μL of water to each well of the ‘Panel B – SamplePrep’ plate.
16. Prepare Reagent D: add 3 mL of MeOH to the Reagent D glass vial and vortex until the solid in the vial is fully dissolved. Add 25 μL of Reagent D to each well of the ‘Panel B – SamplePrep’ plate and shake it at 500 rpm for 30 min.
17. Add 1125 μL of water to the ‘Panel B – L-RA’ tube and vortex for 30 seconds.
18. Add 10 μL of the solution prepared in Step q to each well of the ‘Panel B – LC-MS/MS’ plate except the double blank well.
19. Transfer 25 μL from each well of the ‘Panel B – SamplePrep’ plate to the corresponding wells of the ‘Panel B – LC-MS/MS’ plate and add 215 μL of water to each well. Cover the plate with a silicone mat, shake at 500 rpm for 15 min and transfer the plate to the LC for analysis.

|  | Panel A | Panel B |
| --- | --- | --- |
| Cal 7 | Panel A – LCal7 | Add 100 μL Panel B – LCal7-2 to the Panel B – LCal7-1 |
| Cal 6 | 240 μL Cal 7 + 80 μL H2O | 240 μL Cal 7 + 80 μL MeOH 75% |
| Cal 5 | 160 μL Cal 6 + 80 μL H2O | 160 μL Cal 6 + 80 μL MeOH 75% |
| Cal 4 | 80 μL Cal 5 + 80 μL H2O | 80 μL Cal 5 + 80 μL MeOH 75% |
| Cal 3 | 80 μL Cal 4 + 80 μL H2O | 80 μL Cal 4 + 80 μL MeOH 75% |
| Cal 2 | 40 μL Cal 3 + 160 μL H2O | 40 μL Cal 3 + 160 μL MeOH 75% |
| Cal 1 | 80 μL Cal 2 + 80 μL H2O | 80 μL Cal 2 + 80 μL MeOH 75% |
| QC 3 | Panel A – LQC3 | Panel B – LQC3 |
| QC 2 | 80 μL QC3 + 80 μL H2O | 80 μL QC3 + 80 μL MeOH 75% |
| QC 1 | 80 μL QC2 + 240 μL H2O | 80 μL QC2 + 240 μL MeOH 75% |

**Table S1a**. Calibration and QC standards preparation for Panel A and Panel B.

| **Panel A - Step** |  | **Original Protocol** | **Optimized Protocol** |
| --- | --- | --- | --- |
| 0 | Plate washing | - | 100% MeOH, centrifugation,  N_2_ drying |
| Between d and e | Plate drying | - | N_2_ drying 5 min, 5 LPM |
| p | Extraction-shaking | 300 rpm | 180 rpm |
| q | Plate centrifugation | 50g | 500g |
| s | LC-MS/MS plate shaking | 500 rpm | 300 rpm |
| LC-MS/MS analysis | Injection volume | 10 μL | 15 μL |
| **DFI - step** |  |  |  |
| v | Plate shaking | 500 rpm | 300 rpm |
| **Panel B - Step** |  |  |  |
| f | DBS incubation | Overnight incubation at -20°C | 20 min shaking at 4°C |
| i-I | Reagent A | - | +5s ultrasonic bath |
| i-II | Reagent B | - | +5s ultrasonic bath |
| n | Plate shaking | 500 rpm | 300 rpm |
| p | Reagent D | - | +5s ultrasonic bath |
| p | Plate shaking | 500 rpm | 300 rpm |
| s | Sample dilution | 25 μL+215 μL H_2_O | 50 μL+200 μL H_2_O |
| s | Plate shaking | 500 rpm | 300 rpm |
| LC-MS/MS analysis | Injection volume | 10 μL | 20 μL |

**Table S1b**. Original vs Optimized protocol. The first column indicates the Panel (A, DFI, B) and step of the original protocol that was modified. The second column represents which part of the protocol was modified. Column three and four are the original and the optimized protocol.

**S2.** TMIC MTX MEGA assay characteristics and metabolite classes.

|  | **Panel A** | **Panel B** |
| --- | --- | --- |
| Sample volume | 10 µL | 30 µL |
| Derivatization | PITC | 3-NPH |
| LC-MS | Positive | Negative |
| DFI-MS | DFI 1, DFI 2 | - |

**Table S2a.** Analytical characteristics of Panel A and B, including required sample volume, derivatization, LC-MS ionization polarity and DFI-MS. Abbreviations: PITC – Phenylisothyocyanate, 3-NPH – 3-nitrophenylhydrazine.

| **Panel A** | **Panel B** | **DFI 1** | **DFI 2** |
| --- | --- | --- | --- |
| Alkaloids and derivatives | Amino acids and derivatives | Carnitines | Ceramides |
| Amino acids and derivatives | Fatty acids and derivatives | Hexose | Cholesterol esters |
| Biogenic amines | Indole derivatives | LPC | DC |
| Catecholamines and derivatives | Ketone and keto acids | PC | TG |
| Dipeptides | Nucleobases and nucleosides | SM | HexCer |
| Indole derivatives | Organic acids |  | DiHexCer |
| Nucleobases and nucleosides | Phenolic acids |  | TriHexCer |
| Organic acids | Sugars and derivatives |  |  |
| Others | Sulfates |  |  |
| Sulfates | Trp-Kyn Pathway Metabolites |  |  |
| Trp-Kyn Pathway Metabolites |  |  |  |
| Vitamins and derivatives |  |  |  |

**Table S2b.** Metabolite classes covered by Panel A, Panel B, DFI 1 and DFI 2. Abbreviations: Trp-Kyn – Tryptophan-Kynurenine, LPC – Lysophosphatidylcholines, PC – Phosphatidylcholines, SM – Sphingomyelins, DC – Diacylglycerols, TG – Triacylglycerols, HexCer – Hexosylceramides, DiHexCer – Dihexosylceramides, TriHexCer – Trihexosylceramides.


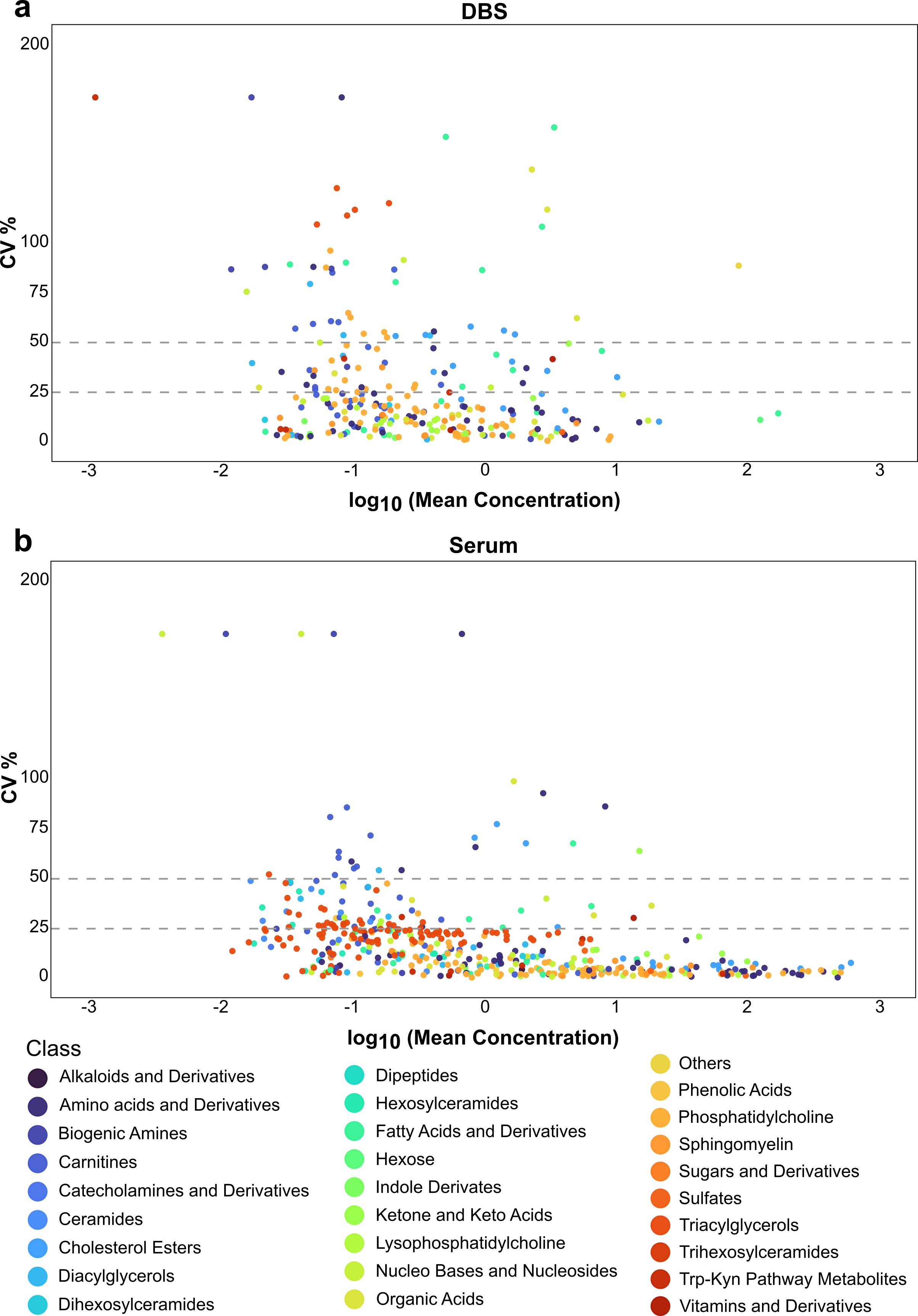


**S3.** Biological variation for DBS (a) and serum (b), considering pool samples. CV% is on the y-axis, log10 (mean concentration) is on the x-axis. The two plots represent the biological variation of all the classes detected (different colors according to the legend) across DBS and serum pools.


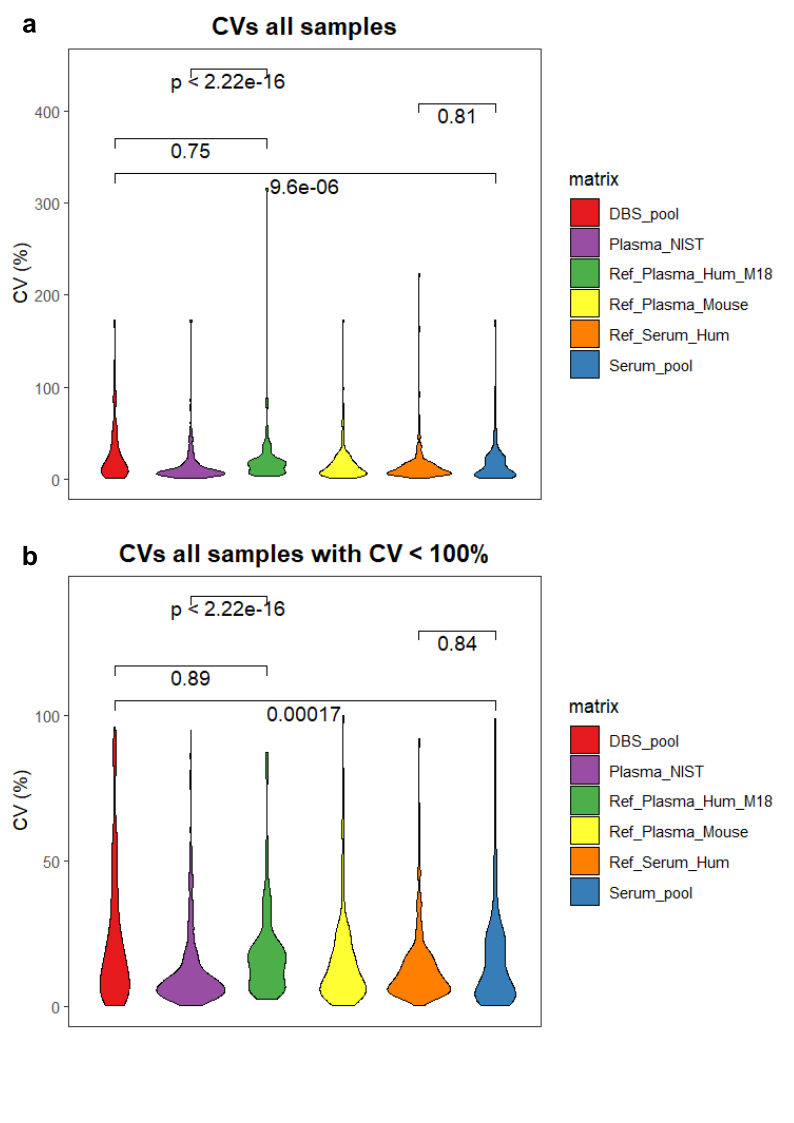


**S4.** Violin plots showing the distribution of coefficient of variation (CV%) across all the evaluated matrices. CV% was calculated across all samples for each metabolite. **a)** The upper plot includes all quantified metabolites. **b)** the lower one shows CV % for all samples considering a threshold of CV < 100%.
